# Neurogasobiology of migraine: Carbon monoxide, hydrogen sulfide, and nitric oxide as emerging pathophysiological trinacrium relevant to nociception regulation

**DOI:** 10.1515/med-2025-1201

**Published:** 2025-05-17

**Authors:** Anastasiia Badaeva, Luigi Maiolino, Andrey Danilov, Margarita Naprienko, Alexey Danilov, Ursula M. Jacob, Vittorio Calabrese

**Affiliations:** Department for Pathological Physiology, I.M. Sechenov First Moscow State Medical University of the Ministry of Health of the Russian Federation, Moscow, Russia; Department of Medical, Surgical Advanced Technologies “G. F. Ingrassia”, University of Catania, Catania, Italy; Department for Nervous Diseases, I.M. Sechenov First Moscow State Medical University of the Ministry of Health of the Russian Federation, Moscow, Russia; Department for Sport Medicines and Medical Rehabilitation, I.M. Sechenov First Moscow State Medical University of the Ministry of Health of the Russian Federation, Moscow, Russia; Institute of Regeneration and Prevention, Healthcare AG, Zurich, Switzerland; Department of Biomedical and Biotechnological Sciences, University of Catania, Via Santa Sofia 97, Catania, 95123, Italy

**Keywords:** nitric oxide, carbon monoxide, hydrogen sulfide, nitroglycerin, epigenetic, migraine

## Abstract

**Background:**

Nitric oxide (NO), carbon monoxide (CO), and hydrogen sulfide (H₂S) are bioactive gasotransmitters implicated in migraine pathophysiology. These gases regulate vascular tone, nociceptive transmission, and inflammatory pathways, playing key roles in both the onset and modulation of migraine.

**Methods:**

This review synthesizes current evidence on the role of NO, CO, and H₂S in migraine, focusing on their molecular mechanisms, interactions, and potential therapeutic implications. Data from human and animal studies were analyzed to elucidate their contributions to migraine pathogenesis.

**Results:**

NO is a well-established migraine trigger, with NO donors such as nitroglycerin inducing headache and migraine attacks via cyclic guanosine monophosphate (cGMP)-dependent pathways. CO interacts with NO and cGMP signaling in pain modulation, contributing to central and peripheral nociceptive processing. H₂S exerts dual effects: while its interaction with NO forms nitroxyl (HNO), activating transient receptor potential ankyrin 1 (TRPA1) channels and triggering calcitonin gene-related peptide (CGRP) release; it also demonstrates neuroprotective properties through antioxidant mechanisms and nuclear factor erythroid 2-related factor 2 (Nrf2) activation. Additionally, epigenetic modifications of calcitonin gene-related peptide alpha (CALCA) have been implicated in migraine susceptibility, further supporting the role of these gasotransmitters in disease pathology.

**Conclusion:**

The interplay between NO, CO, and H₂S represents a critical aspect of migraine pathophysiology, influencing vascular, inflammatory, and nociceptive pathways. Understanding these gasotransmitters’ roles may provide novel therapeutic targets for migraine management, particularly through modulation of TRPA1-CGRP signaling and oxidative stress pathways. Further research is warranted to explore their clinical applications in migraine treatment.

## 1 Introduction

Migraine is a complex and debilitating neurological disorder characterized by recurrent headache attacks often accompanied by sensory disturbances, photophobia, phonophobia, cognitive impairment, nausea, and vomiting. With a global prevalence of approximately 15%, migraine poses a significant public health burden, impacting both individual quality of life and societal productivity. Despite substantial research, the precise pathophysiological mechanisms underlying migraine remain incompletely understood, involving a multifaceted interplay of environmental, neurobiological, and genetic factors [1–4].

Several key mechanisms have been implicated in migraine pathogenesis, including cortical spreading depression (CSD), neurogenic inflammation, oxidative stress, vascular dysfunction, and activation of the trigeminovascular system [5,6]. These processes contribute to heightened neuronal excitability, peripheral, and central sensitization, and dysregulation of pain-processing pathways. In recent years, gaseous signaling molecules – also known as gasotransmitters – have emerged as critical modulators of migraine-related nociception and vascular regulation.

Among these, nitric oxide (NO), carbon monoxide (CO), and hydrogen sulfide (H₂S) play crucial roles in the central nervous system, influencing vasodilation, neuroinflammation, oxidative stress response, and synaptic plasticity [7–9]. The role of NO in migraine is well-established, with NO donors such as nitroglycerin (NTG) known to induce migraine attacks via activation of cyclic guanosine monophosphate (cGMP)-dependent pathways. However, the contributions of CO and H₂S to migraine pathophysiology remain less explored despite emerging evidence suggesting their involvement in pain modulation and neurovascular function.

This review aims to synthesize current knowledge regarding the pathophysiological relevance of NO, CO, and H₂S in migraine. By examining their molecular interactions, neurovascular effects, and potential therapeutic implications, we provide a comprehensive perspective on their roles in migraine mechanisms and identify promising directions for future research.

## 2 Epigenetics of migraine

Epigenetics refers to variations in gene expression and function that do not affect the nucleotide sequence of DNA. This process, through the regulation of individual gene expression, can mediate cell-cycle development [10]. DNA methylation is considered one of the most crucial types of epigenetic modifications, and it has been implicated in various neurological diseases, such as migraines and depressive disorders. Evidence suggests that alterations in the DNA methylation patterns of genes related to migraines, including calcitonin gene-related peptide alpha (CALCA) and receptor activity modifying protein 1 (RAMP1), result in aberrant protein expression, ultimately contributing to migraine [11,12]. The CALCA and RAMP1 genes encode calcitonin-gene-related peptide (CGRP), which is an important factor involved in migraine pathogenesis [13,14].

Although interest in the epigenetics of these migraine-related genes is growing, more detailed studies are needed to uncover the correlation between the methylation patterns of CALCA and RAMP1 and key mechanisms of migraine, as well as clinical and prognostic factors and response to therapy in migraine patients [15]. Additionally, methylenetetrahydrofolate reductase (MTHFR), estrogen receptor 1 (ESR1), and nitric oxide synthase 1 (NOS1), along with their methylation patterns, are considered important genes involved in migraine pathogenesis. The NOS1 gene encodes nitric oxide synthase (NOS), which mediates NO production – a significant factor in migraine pathogenesis [11]. Thus, alterations in NO generation due to epigenetic changes in the NOS gene indicate a significant correlation between NOS1 methylation and the etiology of migraine [16].

Moreover, epigenetic alterations in MTHFR and ESR1 genes have been shown to be relevant to elevated homocysteine plasma levels and reduced mRNA content of estrogen receptor alpha (ERα), respectively, both of which are associated with migraine pathology [16,17]. Additionally, it appears that methylation of the DGKG gene, which encodes diacylglycerol kinase gamma, is closely associated with migraine pathogenesis, although limited evidence currently exists to evaluate the clinical relevance of epigenetic alterations in this gene [15].

In addition to methylation, two other important epigenetic modifications, histone modification and noncoding RNA, are linked to migraine epigenetics. For example, histone H3 trimethylation at lysine 27, an abnormal histone modification, has been shown to be relevant to the expression of pro-inflammatory markers, which contribute to migraine pathogenesis [18]. Furthermore, environmental factors, such as toxicants, stress, and diet, are likely as strongly associated with the development of migraine as genetic factors [19]. Regarding toxicants, DNA methylation changes following exposure to various environmental toxins, such as phthalates – particularly during developmental periods – may contribute to the development of brain diseases [15]. Growing evidence indicates that environmental factors, such as dietary changes, may influence epigenetic modifications, thereby affecting gene expression patterns implicated in migraines [20]. These alterations could potentially alleviate the burden of migraine through neuro-nutritional interventions. However, the precise relationship between epigenetic changes induced by such factors and the onset and progression of neurological disorders remains poorly understood. Consequently, further in-depth studies are required to explore the complex interactions between epigenetics and environmental influences.

Moreover, recent evidence has indicated that H_2_S interacts with various epigenetic processes, such as DNA methylation, which impacts gene expression associated with the development of neurological diseases [21,22]. DNA methylation alterations induced by oxidative stress can lead to decreased antioxidant defense gene expression, increasing the vulnerability of neurons to oxidative damage [23]. It seems that H_2_S regulates epigenetic processes through the activation of oxidative stress and reactive oxygen species (ROS) generation, which play significant roles in neurological diseases like migraines [22]. In addition to DNA methylation, H_2_S also contributes to histone modification and noncoding RNAs, such as microRNAs (miRNAs), whose dysregulation is implicated in neurodegeneration [24,25]. Thus, exploring the interaction between epigenetics, H_2_S, and oxidative stress as mechanisms underlying neurodegeneration offers new insights into epigenetic-based therapies for treating neurological disorders, including migraines.

Furthermore, epigenetic mechanisms can regulate basal synaptic activity, leading to changes in the brain epigenome and subsequent migraine attacks [26]. In 2008, genetic investigations on migraine patients revealed that environmental factors likely depend on epigenetic changes. This finding suggested that epigenetics plays a key role in medication responses. Furthermore, the response to triptans – commonly used in migraine treatment – may change over time in migraine patients, which can be explained by epigenetic alterations in target molecules [27,28].

Overall, in addition to the unsatisfactory response to therapy in migraines, there are other limitations in studies on the clinical features of migraine epigenetics, such as small sample sizes, inconsistent methodologies, and challenges in translating animal model results to human models of migraine. Nevertheless, since epigenetic changes mediate migraine pathogenesis in human models, further understanding of these underlying mechanisms is crucial for discovering new avenues for pharmacological treatment.

## 3 Migraine attack pathogenesis and CGRP

The initiation of a migraine attack begins with the stimulation of peripheral nociceptive neurons innervating the dura mater. This leads to the release of vasoactive peptides, such as CGRP and pituitary adenylate cyclase-activating peptide (PACAP), which further transmit nociceptive signals via the trigeminovascular pathway [29]. CGRP also promotes vasodilation of meningeal blood vessels, increases vascular permeability, and contributes to neuroinflammation [30]. After prolonged activation by these peptides, peripheral trigeminal neurons become sensitized to stimuli from the medulla – resulting in a decreased response threshold and an increased magnitude of the response to stimuli. Peripheral sensitization is responsible for the characteristic throbbing migraine pain, which intensifies with activities like bending and coughing [31].

Studies have shown that CGRP release plays a significant role in the initiation and maintenance of peripheral sensitization [32]. Modern CGRP-based drugs have revolutionized migraine treatment by specifically targeting the CGRP pathway, offering relief for many patients [33–35]. However, migraines are complex neurovascular disorders involving multiple biochemical pathways [36]. In genetically predisposed individuals, migraine triggers can increase neuroinflammation, oxidative and nitrosative stress, reduce ATP and glycogen levels in the brain, and disrupt gas balance [37,38]. These processes trigger a cascade of events that aim to restore energy homeostasis in brain mitochondria. However, this simultaneously favors the development of cortical depression, activation of the trigeminal–vascular system, and the release of CGRP, PACAP, and the opening of ATP-sensitive potassium channels [39].

Emerging evidence highlights the significant roles of gasotransmitters – NO, H₂S, and CO – in migraine pathogenesis [40]. These molecules influence vasodilation, nociception, and neuroinflammation, making them attractive targets for novel therapies [41].

The development of drugs targeting these pathways is a critical step toward addressing unmet needs in migraine treatment. By expanding beyond CGRP, future therapies could offer relief for patients with refractory migraines, improve outcomes through multi-pathway targeting, and support personalized approaches to care. Innovative research in this area holds the potential to close the gap in migraine management, paving the way for a new era of comprehensive and effective treatments.

## 4 NO model of migraine

The first evidence regarding the role of NO in headache attacks was discovered many years ago, suggesting that it may be involved in the pathogenesis of migraines [8]. NO is generated in cells by the oxidation of l-arginine, an essential substrate for NO formation, into l-citrulline through the action of a family of enzymes called NOSs [42,43]. Therefore, NOSs are involved in the endogenous production of NO via their isoforms, including inducible NOS (iNOS), endothelial NOS (eNOS), and neuronal NOS (nNOS), which are homologous but have distinct functional activities. Among these isoforms, eNOS and nNOS are calcium/calmodulin-dependent, with a low output of NO, whereas iNOS is calcium-independent and generates a high level of NO [44].

Since NO is considered an important factor in migraine, a key question is where its action might occur. This is relevant to the activation of three potential sites: NO production in nitroxidergic nerves, vascular endothelium, and the central nervous system [45,46]. Thus, it is possible that the induction of these mechanisms, resulting in NO production, could play a significant role in migraine. Extensive research has shown a positive correlation between NO and various primary headache types, including migraine, tension-type headache, and cluster headaches, highlighting the importance of NO in generating and maintaining headache attacks [8,44].

Additionally, the release of NO as a triggering molecule for migraines can be stimulated by the activation of several membrane-bound receptors, including 5-hydroxytryptamine, histamine, bradykinin, acetylcholine, endothelin-1, glutamate, and CGRP. Apart from NO, CGRP has been recognized as an important mediator in the pathophysiology of migraine. However, the question of whether and how these molecules are linked to migraine pathogenesis remains not fully understood [47]. Furthermore, binding of NO to soluble guanylate cyclase (sGC) activates the enzyme, leading to increased levels of cyclic guanosine monophosphate (cGMP), which in turn induces the opening of large conductance calcium-activated potassium (BKCa) and ATP-sensitive potassium (K_ATP_) channels, both of which are involved in migraine attacks [48,49]. On the other hand, inhibition of cGMP reduction leads to the provocation of migraines, indicating that cGMP plays a significant role in NO-induced migraines. Therefore, targeting one or more steps in the NO-cGMP pathway may offer potential avenues for developing new drugs for migraine and other headache treatments [46].

## 5 *In vivo* studies of NO mechanisms in migraine

The neurophysiological functions of NO have been studied in preclinical models, which are crucial for understanding the mechanisms involved in migraine and developing effective treatments [50,51]. Several studies have investigated the use of NTG (also known as glyceroltrinitrate or GTN) to mimic NO in generating migraine models in both humans and animals [47,52–54]. NTG activates migraine-like attacks by inducing NOS and increasing NO production [46,55]. Although the exact mechanism of NTG activation and NO release remains unclear, it seems that mitochondrial aldehyde dehydrogenase (ALDH-2) mediates the reduction of NTG to NO [56,57].

Initial studies in animal models showed that subcutaneous (SC) injection of NTG (10 mg/kg) in rats induced neuronal activation in various brain regions involved in the biology of migraine, such as the trigeminal nucleus caudalis (TNC) [58]. It also led to an increase in NOS activity in the TNC region [59]. Intravenous (IV) NTG administration induced mild-to-moderate throbbing and biofrontal-like headaches in both migraine patients and healthy subjects, suggesting that NTG is a reliable model for migraine and vascular headaches [60]. It seems that the headaches related to the vasodilatory effects of NTG are mediated by its conversion to NO in the vascular endothelium [61]. However, whether the effects of NO in migraine involve vasodilation remains inconclusive [62].

Furthermore, after NTG administration into the TNC region, the release of CGRP, nNOS, substance P, and subsequent vasodilation were increased, suggesting that the activation of the NO system induces the release of vasoactive neuropeptides from perivascular nerve terminals [63]. NTG can be used in both episodic migraine and chronic migraine models, with a single injection inducing episodic migraine and multiple injections inducing chronic migraine [64]. In a rat model of chronic migraine, infusion of NTG led to the development of photophobia and allodynia, which supports the development of migraine symptoms. Moreover, NO demonstrated pro-nociceptive firing in the trigeminal meningeal afferents, which was not dependent on purinergic receptor P2X subtype 3 (P2X3) and transient receptor potential vanilloid subtype 1 (TRPV1) activities [64].

P2X3 and TRPV1 receptors, which are involved in nociceptive signal generation and mechanical pain modulation, respectively, are important in migraine mechanisms [65,66]. Additionally, migraine-like periorbital and hind-paw hypersensitivity was observed after acute NTG infusion (10 mg/kg) in conscious mice [67]. Chronic infusion of NTG (10 mg/kg, 5 times over 9 days) induced more frequent migraine attacks in subjects. NTG also mediated persistent periorbital and hind-paw hypersensitivity 6–7 days following the last NTG administration [68,69]. Although NTG is recognized as a reliable migraine model, it is not without limitations. Some subjects fail to respond to NTG, and there are higher rates of endothelial cell proliferation and structural differences in the vascular endothelium, which is responsible for converting NTG into NO in mice, compared to humans and rats [70].

As mentioned earlier, the interaction between CGRP and NO supports the role of NO in migraine, and the TRPV1 receptor is thought to facilitate this mechanism. However, clinical evidence does not support the role of TRPV1 blockade in migraine, and CGRP may activate vasodilation via NO production [71–74]. This bidirectional interaction between CGRP and NO is likely crucial in the pathophysiology of migraine.

## 6 Clinical studies for NO mechanisms in migraine

The ability of NTG to induce a migraine-like headache, often resembling spontaneous migraine attacks, makes it the most reliable and reproducible clinical model to investigate the mechanisms involved in migraine [56]. Importantly, NTG-triggered migraines, in addition to representing associated symptoms such as nausea and photophobia, mediate the endogenous release of CGRP in blood plasma samples as a significant migraine mechanism [75].

Evidence shows a difference in the response to NO donors like NTG between migraine patients and non-migraineurs. Migraineurs are sensitive to NTG and experience an immediate headache that persists for several hours, with a latency of 4–6 h. However, non-migraineurs either experience no attack or suffer a short-lasting, rapid-onset headache with mild to moderate intensity [8]. The latency between NTG infusion and the development of migraine headaches suggests that NO initiates slow processes that are involved in the onset of a migraine attack.

Migraine patients who are more sensitive to NTG experience more severe and longer-lasting headaches than healthy volunteers [55]. NG-monomethyl-l-arginine (l-NMMA), a non-selective enzyme inhibitor involved in NO production, inhibits NOS in spontaneous migraine attacks and reduces migraine symptoms. This suggests that, in addition to NO, its related mechanisms are active throughout the attack, and antagonists targeting NO or its associated cascade may be effective in migraine treatment [76,77,78]. Therefore, it seems that NO plays a role in both the initiation and maintenance of migraines [44].

To demonstrate that NO is the causative factor in NTG-induced headaches, it was shown that N-acetylcysteine, which facilitates NTG conversion to NO, increased headache attacks and prolonged superficial temporal artery dilation [78,79]. This suggests that NTG likely causes headaches through NO mechanisms.

In migraine patients, NTG infusion triggers severe and persistent attacks of migraine without aura, suggesting that NO is unlikely to be involved in the aura. However, the induction of a migraine aura in two reported subjects following NTG infusion suggests that the aura might occur randomly [80–83].

The administration of sildenafil, a cGMP-hydrolyzing phosphodiesterase-5 inhibitor, increased cGMP levels and induced migraine attacks in 10 out of 12 migraine patients without aura. This demonstrated that not only NO itself but also the increase in cGMP through NO or other mediators plays an important role in migraine induction [84].

Levels of arginine, citrulline, ornithine, monomethyl arginine (MMA), and asymmetric dimethylarginine (ADMA), endogenous NOS inhibitors, as well as symmetric dimethylarginine (SDMA), an inactive stereoisomer of ADMA that may compete for arginine uptake by cells, were measured in interictal chronic migraine patients and healthy subjects [85]. Patients with chronic migraine had low plasma arginine levels and elevated levels of ornithine, ADMA, and MMA, while citrulline and SDMA levels were the same as in controls. This indicates that arginine metabolism is impaired in chronic migraine patients, and elevated levels of ADMA and MMA reduce the amount of circulating NO. However, another study found that plasma arginine levels were significantly higher in patients with episodic migraine compared to those with chronic migraine [86]. In addition, ADMA and SDMA levels were also significantly higher in migraine patients than in controls. Arginine levels were positively associated with the level of pain experienced by migraine patients. Citrulline levels were also reduced in patients with episodic migraine as part of a metabolomic screening [41]. This suggests that migraine attacks require enhanced NO production via NOS-induced conversion of arginine to citrulline.

In conclusion, evidence indicates that NO is a crucial element in migraine pathogenesis, either independently or via mediating a nitrergic pathway that involves other neurobiochemical processes. Targeting elements of this cascade may represent a potential therapeutic approach for migraine treatment.

## 7 CO model of migraine

The second gasotransmitter, CO, is well known as a silent killer gas due to its colorless, odorless, and toxic effects. Despite its toxic properties, CO is considered an endogenous molecule with various physiological and biological functions [7,87–89], ranging from the regulation of microcirculation, inflammation, mitochondrial homeostasis, neurotransmission, and motility [90]. Endogenously, CO is generated as a byproduct of heme catabolism through heme-oxygenase (HO) activity, which exists in two major isoforms: HO-1 (inducible) and HO-2 (constitutive) [91]. Recent studies highlight the pivotal role of HO-2 in pain regulation, particularly in the spinal cord [92]. Additionally, CO influences prostaglandin synthesis at the hypothalamic level, which may have implications for migraine pathophysiology [93].

Interestingly, CO, as a pain-modulating neurotransmitter, can be involved in pain transmission through cGMP pathways, which likely play a significant role in the pathophysiology of migraines and other headaches [7]. cGMP also activates cGMP-dependent ion channels, protein kinases, and phosphodiesterases, which regulate various intracellular functions [7,46]. One of the important clinical effects of CO is headache [77], as supported by several studies [7,94,95].

According to the International Classification of Headache Disorders (ICHD-III beta), CO-induced headache is described as a bilateral headache, with intensity depending on the severity of intoxication. This headache is usually frontal at moderate to severe stages and shares many features with migraine, including nausea and vomiting, which are common symptoms and aggravated during physical activities [96,97]. CO binds to heme, producing carboxyhemoglobin (COHb) with an affinity 210–250 times higher than oxygen, leading to a reduction in oxygen-carrying capacity, impaired oxygen release, and eventually tissue hypoxia [98]. Thus, the combination of COHb production and direct CO effects at the cellular level likely plays a significant role in the clinical effects of CO.

In a clinical study, following CO exposure (mean COHb 20%), cerebral blood flow (CBF) increased by 26% due to a hypoxia-induced compensatory reaction. However, the response of CBF to CO in humans is variable and not well associated with COHb [94]. *Ex vivo* studies have indicated that CO could induce cerebral and systemic artery dilation, and since cerebral vessel dilation occurs during spontaneous migraine attacks, this suggests that CO may be a crucial molecule in primary headaches [99]. On the other hand, the vascular action of CO may also depend on its interference with NO [95].

Hypoxia was previously considered the sole mechanism involved in CO-induced headaches [94,100], but recent data suggest that CO-induced headaches may also occur following non-toxic doses of exposure. According to animal pain model studies, CO can act as a pain-modulating neurotransmitter through interactions with NO, glutamate, activation of cGMP pathways, oxidative stress, and regulation of the expression of nociception-related genes [7,101]. This finding supports the idea that CO may be significantly involved in migraine and other headache mechanisms.

As mentioned earlier, activation of the cGMP pathway is likely a crucial element in migraine and other headache pathogenesis, and CO, through cytoplasmic guanylate cyclase (sGC) stimulation, increases cGMP production, potentially leading to headache production. Interestingly, CO and NO mechanisms share high similarities, as both dilate cranial vessels and increase cGMP production in relation to primary headaches such as migraine. Thus, the role of CO in migraine production can be supported through its interaction with NO, which is an important molecule in migraine and other headaches. Moreover, further investigation into the effects of NO and its mechanisms involved in migraines could provide further insight into CO mechanisms in headaches [83,102].

However, findings from a clinical model demonstrated that CO could not provoke more migraines in migraine patients compared to healthy individuals, though it could induce more headaches in patients. In this study, 50% of the migraine patients experienced migraine-like attacks after CO exposure, compared to 80% of the patients who received NTG as a NO donor. This suggests that CO may not have potent effects and is not as effective as NO in inducing migraines [70,103]. Furthermore, CO results in relatively mild hypoxia by reducing hemoglobin’s oxygen-binding capacity, which may explain the lower potency of CO in inducing migraines.

The study results also indicated that CO, at non-toxic concentrations, had low potency in inducing migraines, suggesting it is not a suitable model for investigating migraines. Various factors could explain the difference in potency between NO and CO in inducing migraines: (1) NO has a greater potent effect on cGMP activation compared to CO [104], (2) CO’s strong binding to hemoglobin results in a small amount of free CO available for linking to sGC, and (3) the limitation on using higher concentrations of CO due to its toxicity.

Moreover, in another human model, only one healthy volunteer experienced a migraine-like attack following CO exposure, compared to six migraine patients [94]. In a human headache model, short, intense CO inhalation induced mild, prolonged headaches without vasodilation of the radial and superficial temporal arteries in normal volunteers. In this study, 12 healthy volunteers received either CO or placebo on two study days. Ten volunteers developed headaches after CO exposure, compared to six after a placebo. This may be related to hypoxic and direct cellular effects, including cGMP pathways and NO (cGMP-NO) interactions [84].

Additionally, several studies have reported that chronic exposure to CO at non-lethal concentrations led to migraine-like attacks, and migraine symptoms were eliminated once the CO source was removed [94]. In another case–control study, the NO and CO concentrations in paranasal sinus air were compared between migraine patients and healthy subjects. The results showed that NO concentrations above 50 ppb were present in the sinus air of migraine sufferers, whereas in normal subjects, NO was below 50 ppb. Similarly, CO levels above 1 ppm were observed during acute migraine episodes, higher than in healthy individuals. These results support the idea that high NO and CO levels in the paranasal air of migraine patients may be directly associated with migraine presence. Thus, NO and CO concentrations in the paranasal air may be used to distinguish between migraine patients and healthy individuals, especially when NO concentrations above 50 ppb may serve as a diagnostic model for migraine [55].

**Figure 1:**
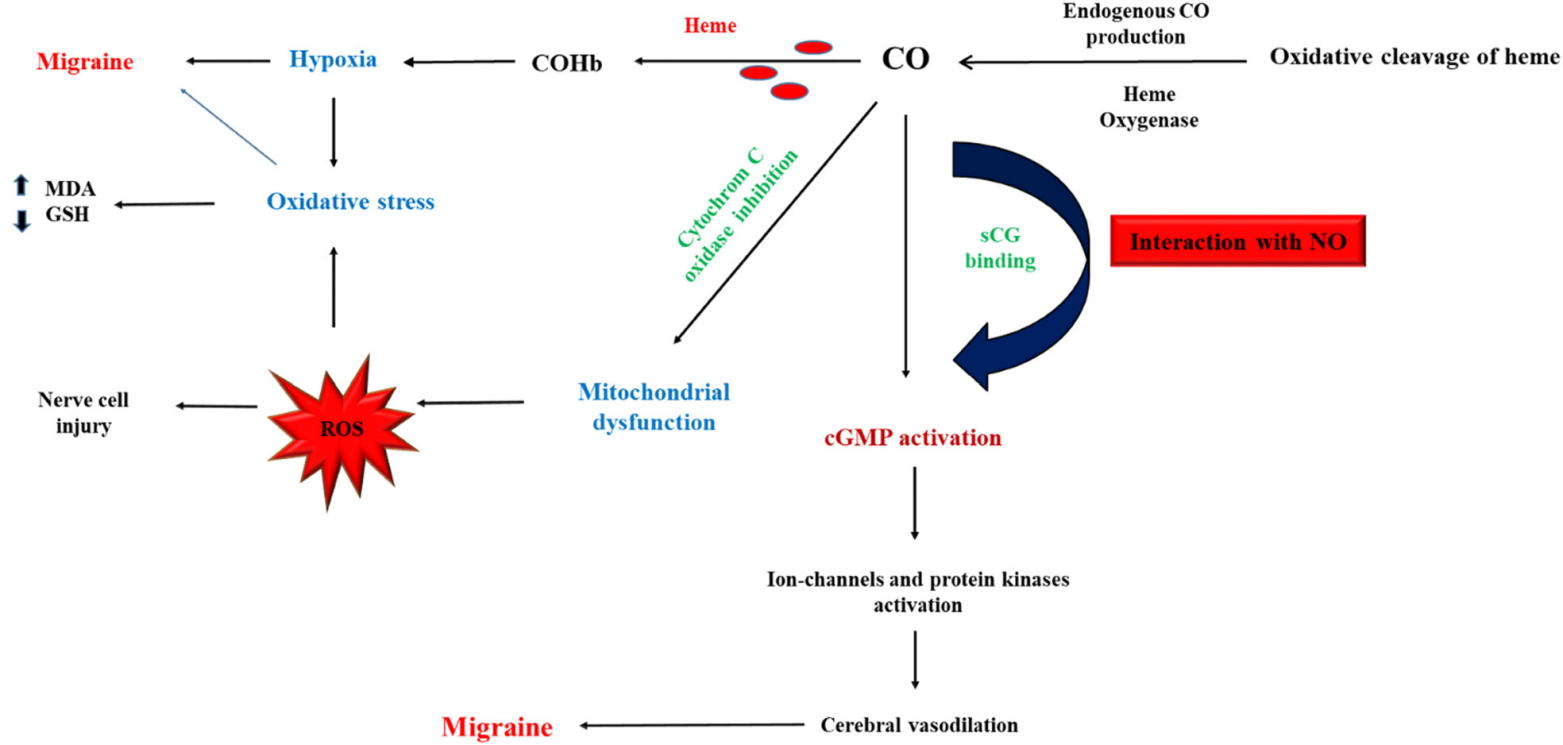
The central role of CO in migraine pathogenesis and its interaction with NO and other processes related to migraine. CO binds to the heme-producing carboxyhemoglobin (COHb), leading to tissue hypoxia and oxidative stress, which increases the levels of malondialdehyde (MDA) and GSH depletion, suggesting induced migraine pathogenesis. CO induces mitochondrial dysfunction, leading to ROS production and oxidative stress. CO acts as a pain-modulating neurotransmitter through interactions with NO and activation of cyclic guanosine monophosphate (cGMP) pathways.

Oxidative stress, according to experimental models of migraine, appears to be involved in migraine pathogenesis, though the primary mechanism is not fully understood [105]. Studies have demonstrated that exposure to CO at toxic concentrations leads to oxidative stress and ROS, resulting in lipid peroxidation and glutathione (GSH) reduction, which contribute to the development of neurological pathology [106]. On the one hand, inhibition of cytochrome *c* oxidase (CcO), a crucial element in mitochondrial oxidative metabolism through CO, may contribute to oxidative stress and ROS production, inducing migraine-like attacks. On the other hand, activation of the cGMP pathway by CO likely induces oxidative stress and cellular damage [7]. Therefore, the role of CO in migraine pathogenesis through various mechanisms has been supported in several studies, as summarized in Figure 1, which outlines all the key mechanisms involved.

## 8 H_2_S model of migraine

H_2_S is a toxic gas produced through both natural and artificial sources. However, at low concentrations, it functions as a gaseous signal molecule involved in central nervous system diseases [40]. Endogenous H_2_S is predominantly generated from cysteine and homocysteine via enzymatic pathways, including cystathionine γ-lyase (CGL), cystathionine β-synthase (CBS), and 3-mercaptopyruvate sulfur transferase (MPST) in mammalian tissues [107]. H_2_S has been proposed as the third gasotransmitter, alongside CO and NO, and has various physiological functions, including neuromodulatory activities in the nervous system [89]. Several studies have reported that H_2_S and NO effects are mutually interdependent [108–110].

**Figure 2:**
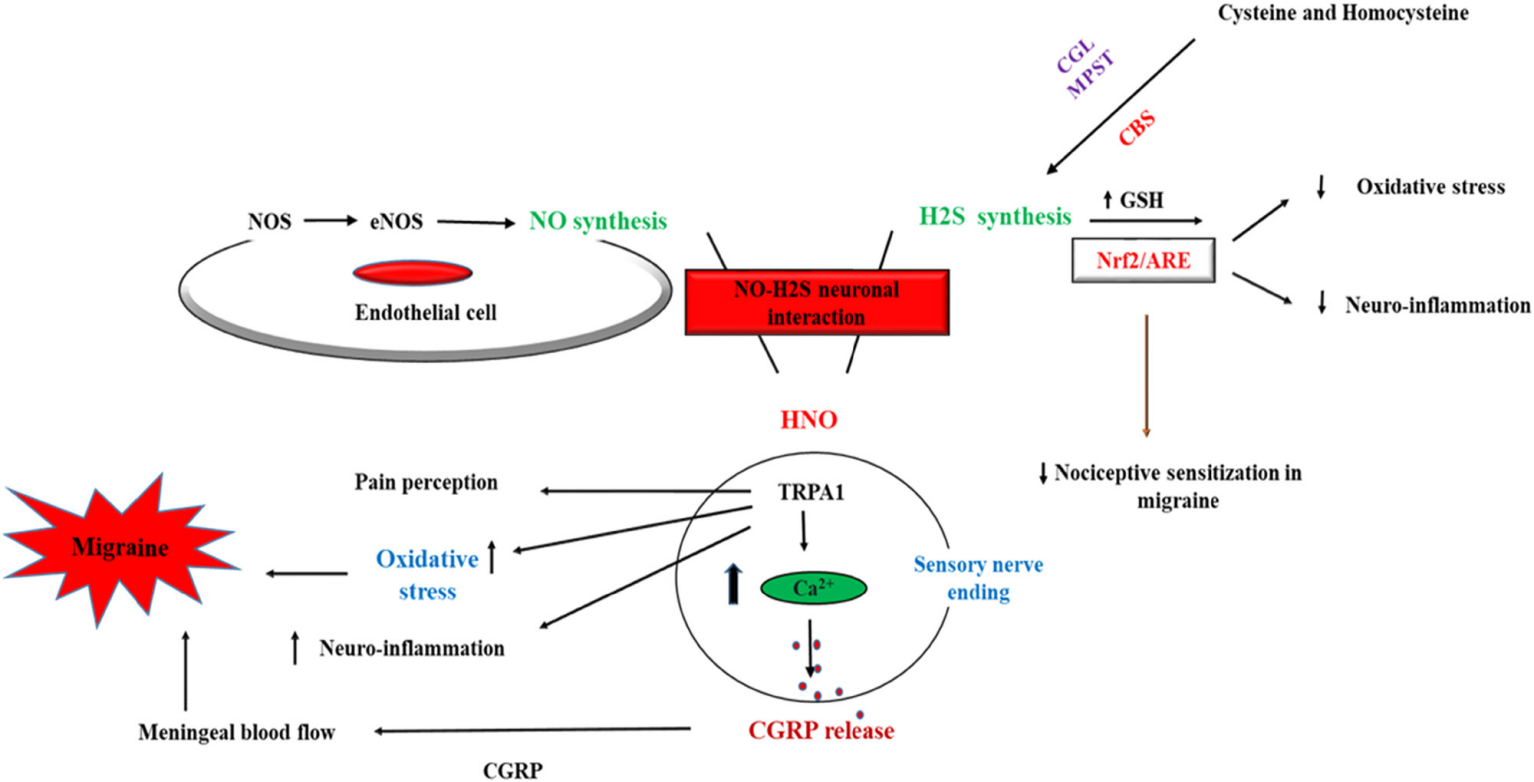
Role of the H_2_S–NO–HNO–TRPA1–CGRP pathway as a crucial element in migraine pathogenesis. The anti-nociceptive effects of H_2_S also is shown. Endogenous H_2_S is predominantly generated from cysteine and homocysteine via enzymatic pathways, including cystathionine γ-lyase (CGL) and cystathionine b-synthase (CBS), as well as 3-mercaptopyruvate sulfur transferase (MPST). NOSs are involved in NO production endogenously via its isoforms, such as eNOS. The interaction of H_2_S and NO results in nitroxyl (HNO) formation, which leads to the activation of the TRPA1 channels, causing an influx of Ca^2+^ and release of calcitonin gene‐related peptide (CGRP), inducing meningeal blood flow and migraine. TRPA1 affects oxidative stress and neurogenic inflammation, causing migraine induction. H_2_S exerts its neuroprotective effects via increasing GSH and targeting nuclear factor erythroid 2-related factor 2 (Nrf2)–antioxidant response element (ARE) signaling pathway to reduce the oxidative stress and neuroinflammation, and eventually nociceptive sensitization reduction in migraine.

The interaction between H_2_S and NO results in the formation of nitroxyl (HNO), a redox sibling of NO, which activates the transient receptor potential ankyrin 1 (TRPA1) channels. This activation leads to the release of CGRP from primary afferents, inducing meningeal blood flow [111]. According to vascular and neuroinflammatory migraine theories, meningeal artery dilation is considered a significant mechanism in headache generation. Neurogenic vasodilatation, related to CGRP release as a potent vasodilator, can thus lead to the induction of primary headaches, such as migraines. This supports the efficient role of CGRP in migraine induction via the HNO-TRPA1-CGRP signaling pathway (Figure 2) [108].

Furthermore, TRPA1 channels, which are active in trigeminal afferents, are co-localized with CGRP. Evidence has demonstrated that activation of TRPA1 channels in the nasal trigeminal afferents is crucial in headache generation caused by inhaled irritants. Therefore, TRPA1 channel activation via HNO, resulting from the interaction of H_2_S and NO, may be highly involved in headache induction mechanisms [112,113]. Additionally, TRPA1 activation may contribute to oxidative stress, a potential factor in migraine pathogenesis [66].

Clinical studies have also shown higher plasma levels of CGRP and NO metabolites during migraines [114]. Increased plasma homocysteine levels, which could indicate elevated H_2_S metabolism, have been reported in migraine patients with aura [115]. These findings suggest that CGRP release may be regulated by HNO, further supporting its role in migraine induction. Moreover, *in vivo* studies have shown that drugs that inhibit CGRP release or its receptor may be effective in treating primary headaches such as migraines [116,117]. The involvement of CGRP in migraine pathogenesis is well-established, and the role of TRPA1 in CGRP release further emphasizes its importance in migraine [66,117].

Since targeting H_2_S and HNO generation is a potential therapeutic approach, further preclinical research is needed to evaluate the effects of the HNO-TRPA1-CGRP cascade in migraine pathogenesis [118,119]. Interestingly, while H_2_S mediates pro-nociceptive firing in trigeminal afferents, it also exhibits anti-nociceptive effects against nociceptive agents in the meninges [120,121]. The pro- and anti-nociceptive effects of H_2_S are likely concentration-dependent. At low doses, H_2_S exerts anti-nociceptive effects, contributing to pain reduction in neurons [122]. This dual role of H_2_S suggests that its effects on migraine may vary depending on its concentration.

Furthermore, the interaction of CBS, the main H_2_S-generating enzyme in nervous tissue, with H_2_S has been shown to exert neuroprotection by increasing cysteine and GSH levels following intracerebral hemorrhage, thus reducing oxidative stress-induced neurodegenerative damage [123,124,125,126]. GSH is crucial for protecting cells from damage triggered by oxidative stress and ROS generation [127]. Several studies have confirmed the neuroprotective effects of H_2_S via its antioxidant properties and its ability to inhibit ROS production [128,129]. Moreover, H_2_S may exert its neuroprotective effects by activating the Nrf2, which protects against oxidative stress and neuroinflammation – key factors involved in migraine pathogenesis [129,130]. Activation of the Nrf2 signaling pathway significantly decreases oxidative stress and neuroinflammation levels [131,132].

The potential role of oxidative stress and neuroinflammation in migraine pathogenesis is increasingly recognized [56,133,134]. Therefore, it seems that H_2_S, by targeting the Nrf2 signaling pathway, may provide protective effects against neurodegenerative diseases induced by oxidative stress and neuroinflammation, such as migraines. Additionally, the protective effects of the Nrf2-ARE signaling pathway have been demonstrated in models of NTG-induced migraines [56,130], suggesting that this pathway could serve as an effective therapeutic target for migraine patients.

## 9 Conclusion

In summary, NO, CO, and H₂S, as potent vasodilators and pain-modulating neurotransmitters, play critical roles in migraine pathogenesis. Through mechanisms such as NO-cGMP activation, hypoxia-related signaling, and the HNO-TRPA1-CGRP pathway, these gases contribute to migraine induction and progression. Current pharmacological approaches targeting these pathways – such as CGRP receptor antagonists and NO inhibitors – offer therapeutic potential, yet a substantial proportion of patients remain refractory to treatment. This highlights the necessity for further research into gasotransmitter-mediated migraine mechanisms to develop more targeted and effective therapies.

Among these gases, H₂S has emerged as a particularly intriguing molecule, given its dual role in migraine pathogenesis. While H₂S interacts with NO to form HNO, a potent TRPA1 activator leading to CGRP release and migraine onset, it also exhibits neuroprotective properties by reducing oxidative stress and neuroinflammation via Nrf2 activation. Despite these insights, the HNO-TRPA1-CGRP signaling cascade remains largely underexplored, warranting further investigation to delineate its precise contribution to migraine. Additionally, epigenetic regulation – particularly the involvement of CALCA and RAMP1, which encode CGRP and its receptor – suggests that gasotransmitter interactions may have broader implications for migraine heritability and chronicity.

Migraine, as a highly prevalent primary headache disorder, is increasingly recognized as a condition driven by oxidative stress, inflammatory pathways, and genetic susceptibility. ROS formation, while often linked to cellular damage, also plays a role in highly regulated stress response pathways, including longevity networks and vitagene signaling. Vitagenes, which encode proteins such as heat shock proteins (Hsp32, Hsp70), γ-glutamylcysteine (γ-GC) synthase, thioredoxin, and sirtuins, serve as key regulators of cellular homeostasis and nociceptive modulation. Given the broad cytoprotective and anti-inflammatory properties of the heat shock response, there is growing interest in identifying pharmacological inducers of these pathways to mitigate migraine-related neuroinflammation and pain sensitization.

This review provides a comprehensive synthesis of gasotransmitter involvement in migraine and highlights knowledge gaps regarding CO and H₂S. While this work primarily focuses on NO, CO, and H₂S, additional gasotransmitters such as histamine and PACAP have been implicated in migraine pathophysiology and warrant further exploration. Furthermore, since epigenetic modifications contribute to migraine susceptibility, future research should aim to identify epigenetic targets that could enhance pharmacological migraine treatment. Ultimately, advancing our understanding of gasotransmitter-mediated mechanisms in migraine may pave the way for novel therapeutic strategies aimed at modulating oxidative stress, neuroinflammation, and nociceptive signaling.
